# Clinical experience, infection control practices and diagnostic algorithms for poxvirus infections - an Emerging Infections Network survey

**DOI:** 10.1186/1756-0500-3-46

**Published:** 2010-02-25

**Authors:** Christine M Hughes, Edith R Lederman, Mary G Reynolds, Inger K Damon, R Ryan Lash, Susan E Beekmann, Philip M Polgreen, the Infectious Diseases Society of America's Emerging Infections Network

**Affiliations:** 1 Centers for Disease Control and Prevention, National Center for Zoonotic, Vector-borne, and Enteric Diseases, Division of Viral and Rickettsial Diseases, 1600 Clifton Road, NE Atlanta, Georgia, 30333, USA; 2 Centers for Disease Control and Prevention, Epidemic Intelligence Service, Office of Workforce and Career Development, 1600 Clifton Road, NE Atlanta, Georgia, 30333, USA; 3 University of Iowa Carver College of Medicine, Department of Internal Medicine, Iowa City, Iowa, 52242, USA

## Abstract

**Background:**

In order to determine how best to tailor outreach messages about poxvirus diagnosis and infection control for health practitioners, we surveyed infectious disease physicians in the Infectious Diseases Society of America's Emerging Infections Network.

**Findings:**

Surveys consisting of two unknown case scenarios designed to raise suspicion for monkeypox and orf were distributed to the 1,080 members of the EIN. The surveys contained questions pertaining to which diagnostic tests, points of contact, and transmission precautions they would likely utilize during patient evaluation. Basic response rates and frequencies of responses were calculated. Comparisons of the survey responses were made using the chi-square test. Of the 212 members who responded (20% response rate), significantly more respondents indicated that they would request diagnostic testing in the context of the monkeypox case scenario as compared to the orf case scenario. A significantly higher number of respondents indicated they would institute droplet or airborne precautions for the monkeypox case as opposed to the orf case scenario.

**Conclusions:**

This survey provided an opportunity for public health practitioners to gain insight into physician approaches to evaluation, diagnosis and reporting of suspected poxvirus-associated infections. This survey identified key areas in which public health practitioners can better serve physicians by focusing on education. As a result we were able to identify potential knowledge gaps and deficits in the availability of useful resources to facilitate accurate case identification and management.

## Findings

In the wake of the 2003 U.S. monkeypox outbreak and renewed concerns regarding bioterrorism, poxvirus infections have garnered increased attention from medical and public health professionals alike.

There are multiple poxviruses of significance to human health that occur in the United States. These include *Molluscum contagiosum virus*, which causes common viral infections of the skin, and various parapoxviruses, such as *Orf virus *and *Pseudocowpox virus*, which are zoonotic entities associated with domestic ruminants (e.g., sheep, goats, cattle). Human parapoxvirus infections occur primarily in rural communities, but may also occur in larger communities with live animal markets, petting zoos and small-scale animal husbandry [1]. Inadvertent *Vaccinia virus *infections also occur in the United States. Vaccinia is the primary component of the smallpox vaccine and infections can occur following contact with a recent vaccinee or via exposure to the virus in a laboratory [2-4]. Additionally, the current oral rabies vaccine (ORV), used to prevent the spread of terrestrial rabies along the Eastern seaboard, consists of a recombinant vaccinia virus containing the rabies virus glycoprotein gene. Two human infections following contact with ORV has been reported [5-7].

Importation of poxviruses from abroad is also a concern. This occurred in 2003 when monkeypox infected African rodents were imported to the United States resulting in a outbreak of monkeypox [8,9]. *Monkeypox virus *is a communicable orthopoxvirus which can cause systemic infections in humans similar to *Variola virus *(smallpox). This event marked the first time that human monkeypox infections had been observed in the Western Hemisphere. There have also been two reports of *Tanapox virus *(a yatapoxvirus) infections in travelers returning to the U.S. from Africa [10,11]

Many poxvirus infections share common clinical features (e.g., vesiculo-pustular or nodular rash lesion characteristics) but have differing risks for person-to-person transmission, thus necessitating different infection control measures. Poxvirus infections can be confused with other infections or conditions. This underscores the importance of laboratory diagnostic evaluation when poxvirus infections are suspected. Diagnostic testing for poxvirus infections is available; however most are only available at specialized reference centers (e.g., Laboratory Response Network facilities, Centers for Disease Control and Prevention (CDC)).

We surveyed infectious disease physicians in the Infectious Diseases Society of America's (IDSA) Emerging Infections Network (EIN) to gather insight on current diagnostic and infection control practices for suspect poxviruses in order to tailor outreach messages to health practitioners. In addition, we sought to determine what resources were readily available to physicians to assist in evaluation of suspected poxvirus infections. This survey also allowed us to gain some insight into the frequency and spectrum of poxvirus infections seen by this network of consultants.

IDSA's EIN is a provider-based emerging infections sentinel network of adult and pediatric infectious disease consultants. This network was established through a Cooperative Agreement Program Award in 1995 from the CDC [12].

During February and March of 2007, surveys were distributed by e-mail and facsimile to the 1,080 EIN. Participants were encouraged to use any reference material they deemed necessary to complete the query and were allowed to log in and out of the survey if they required more time.

**Figure 1:**
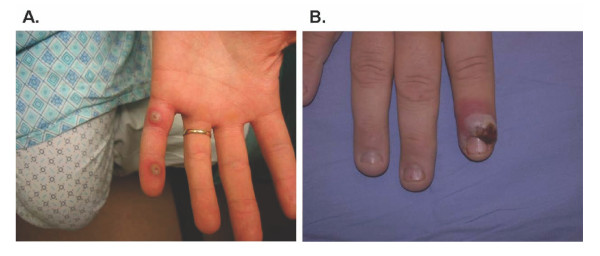
**Case scenario pictures**. **A) Monkeypox case scenario: **23 year old male medical student with several pustular skin lesions (upper and lower extremities including volar surfaces), lymphadenopathy, fever, chills, backache, malaise; he recently returned from Democratic Republic of Congo where he examined patients with undiagnosed febrile rash illness. Photo by Dr. Janet A. Fairley, 2003. **B) Orf case scenario: **42 year old male with two large nonpruritic, painless vesicular lesions on thumb and forefinger; he denies other symptoms, works on farm and recently purchased juvenile goats at auction (some of which had ulcers on their oral mucosa). Photo by Dr. Susan Meidl, 2006

Our survey was developed to assess clinical experience, infection control practices, and diagnostic algorithms related to poxviruses. The survey consisted of two unknown case scenarios designed to raise suspicion for monkeypox and orf (Figure 1A and 1B, respectively) with corresponding questions pertaining to which diagnostic tests and transmission precautions they would likely use during patient evaluation [Additional file 1: Copy of survey]. Members were queried as to their likely immediate points of contact for reporting of the suspicious illnesses. In order to gain some insight on the poxvirus experience for physicians in various regions of the country, members were also asked to document the types of poxviruses they have ever seen in their practice.

Since the first distribution of the survey resulted in a low response rate, respondents were given the option of omitting their name from the second round of distribution. They were also given a choice of submitting their state and practice type in lieu of their name. Therefore, since demographic data is linked by name, some respondents do not have linked demographic data.

Basic response rates for demographic variables and frequencies of specific responses for each survey question were calculated. Denominators varied for several questions as members did not always respond to all the survey questions. Comparisons of responders and non-responders, as well as comparisons in responses to the two case scenarios were made using the chi-square test. A p-value of less than 0.05 was considered statistically significant.

Of the 1,080 EIN members to whom surveys were distributed, 212 (20%) returned completed surveys. Of these 212 surveys, 29 (13.7%) of them were returned without a name and were not able to be linked to corresponding demographic data. Respondents included physicians from all nine U.S. Bureau of Census divisions, along with two respondents from Canada (Table 1). Those with less than 10 years of experience were significantly less likely to respond to the survey compared to those with over 10 years experience (p = 0.02). Those who teach were significantly more likely to respond to the survey then those who don't (p = 0.004). The lowest response rates came from those in an urban setting, those who do not teach, and those with less than 10 years of experience. EIN members from the New England and Mid Atlantic region were the least likely to respond to the survey, while those from the East North Central, West South Central, and the Mountain region were the most likely to respond.

**Table 1:** Geographic and practice characteristics of poxvirus survey respondents vs. entire EIN participant base

Variable	Respondents (n = 212), no.(%)	Total EIN (n = 1076^†^), no.(%)	Response rate
**Type of practice**			
Adult	141 (77.5%)	786 (73.1%)	17.94%
Pediatric	34 (18.7%)	213 (19.8%)	15.96%
Adult & Pediatric	7 (3.9%)	75 (6.97%)	9.33%
Other	0	2 (0.18%)	
			
**Practice Location**			
Rural	11 (7.5%)	48 (6.8%)	22.92%
Suburban	40 (27.4%)	150 (21.3%)	26.67%
Urban	93 (63.7%)	496 (70.6%)	18.75%
combination	2 (1.4%)	9 (1.3%)	22.22%
			
**Teach**			
Yes	131 (72.8%)	637 (61.6%)	20.57%
No	49 (27.2%)	397 (38.4%)	12.34%*
			
**Practice Type**			
Academic	105 (52.2%)	404 (55.9%)	25.99%
Private	84 (41.8%)	264 (36.7%)	31.82%
Other	12 (6.0%)	54 (7.5%)	22.22%
			
**Region**			
New England	13 (6.3%)	92 (8.6%)	13.83%
Mid Atlantic	28 (13.5%)	196 (18.2%)	14.29%
East North Central	36 (17.3%)	144 (13.4%)	25.00%
West North Central	16 (7.7%)	75 (7.0%)	21.33%
South Atlantic	34 (16.4%)	214 (19.9%)	15.89%
East South Central	12 (5.8%)	49 (4.5%)	24.49%
West South Central	18 (8.7%)	72 (6.7%)	25.00%
Mountain	14 (6.7%)	54 (5.0%)	25.93%
Pacific	35 (16.8%)	160 (14.9%)	21.88%
Canada	2 (1%)	13 (1.2%)	15.38%
Puerto Rico	0 (0%)	6 (0.6%)	
			
**No. yrs practice**			
<10 yrs	9 (8.0%)	74 (16.9%)	12.16%*
10-20 yrs	51 (45.5%)	162 (37.0%)	31.48%
21-30 yrs	38 (33.9%)	147 (33.6%)	25.85%
31+ yrs	14 (12.5%)	55 (12.6%)	25.45%

Note: # of respondents does not equal 21 for some variables due to missing information

^†^Demographic data was available for 1076 of the 1080 EIN participants

* Variable group has significantly lower response rate compared to the rest of the responses for that variable combined

Of the 212 respondents, significantly more, 22%, would rely on clinical diagnosis alone for etiologic determination in the context of the orf case scenario in contrast to the monkeypox case scenario, 3% (p < 0.0001). However, the majority of respondents indicated that they would likely request laboratory testing for determination of poxvirus etiology for both case scenarios. Significantly more respondents indicated that they would likely request polymerase chain reaction (PCR) based diagnostic technologies in the context of the monkeypox case scenario as compared to the orf case scenario (87%, vs.67.9%) (p < 0.001) (Table 2). Significantly more respondents, 66.0%, indicated that they would choose a state or federal lab in the monkeypox case for PCR testing, versus 34.0% for the orf case (p < 0.001).

**Table 2:** Diagnostic ordering preferences for the two case scenarios in the EIN poxvirus survey

		Monkeypox Scenario		Orf Scenario	
**Diagnostic Test**	**Lab utilized**	**#**	%*	**#**	%*

PCR	In-house/local academic institution	61	28.8%	70	33.0%
	State/Federal	140	66.0%	72	34.0%
	Commercial reference lab	25	11.8%	26	12.3%
	In-house/local academic institution	37	17.5%	17	8.0%
Serology	State/Federal	106	50.0%	46	21.7%
	Commercial reference lab	36	32.1%	28	13.2%
	In-house/local academic institution	99	46.7%	67	31.6%
Culture/Histopathology	State/Federal	68	32.1%	23	10.8%
	Commercial reference lab	8	3.8%	6	2.8%

Note: Monkeypox specific PCR, culture and histopathology are currently available at CDC. Most state reference laboratories are able to perform orthopoxvirus generic PCR. Serological testing at CDC is orthopoxvirus generic. Orf specific PCR is currently available at CDC while orf serology is available at an outside lab (Viral and Rickettsial Disease Laboratory, California Department of Public Health, Richmond, CA).

* Percent of total responders. Numbers do not add up to 100% as respondents were able to pick multiple choices.

Significantly more respondents would order a serological test for the monkeypox case scenario (72.6%) than for the orf case (38.7%) (p < 0.0001). The majority indicated that they would likely utilize a state or federal lab for serologic testing in the context of the suspected monkeypox case (50%) and the suspected orf case (21.7%). A significantly higher number of respondents would also order a culture or histopathology for the monkeypox case (73.6%) in contrast to the orf case (43.9%) (p < 0.0001). An in-house or local academic lab was most likely to be picked for both case scenarios (46.7% and 31.6% respectively).

**Figure 2:**
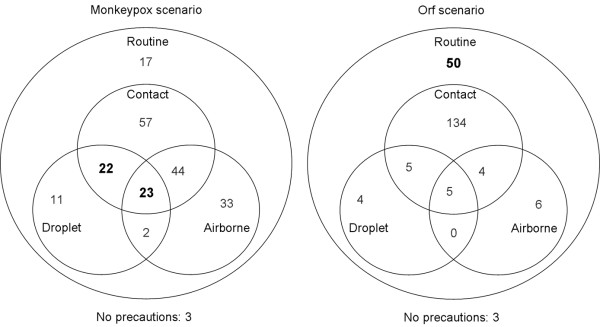
**Likely precautionary measures indicated for each hypothetical case scenario**. The numbers in each portion of the diagram represent the number of respondents choosing that combination of precautions. The appropriate choices have been bolded. CDC recommends a combination of standard, contact, and droplet precautions for possible monkeypox virus (or other systemic orthopoxvirus) infections. In addition, because of the theoretical risk of airborne transmission, Airborne Precautions should be applied whenever possible. CDC recommends standard precautions for possible orf virus infections.

When asked what type of precautionary measures they would likely institute during examination of the patient's described in hypothetical scenario A or B, a significantly higher proportion of respondents indicated that they would choose routine only precautions for the orf case scenario (23.7%), as opposed to the monkeypox case scenario (8.0%) (p < 0.0001) (Figure 2). A significantly higher number of respondents indicated they would institute droplet or airborne precautions for the monkeypox case (22.6% & 48.1% respectively), as opposed to the orf case scenario (6.6% & 7.1%) (p < 0.0001).

When respondents were asked whom they might report initial suspicion of poxvirus-associated illness, a significantly higher proportion of respondents indicated that they would report the suspected monkeypox case (88.2%) as opposed to the orf case (66.8%) (p < 0.0001), with most respondents choose the state or local health department as the first point of contact for either scenario.

**Figure 3:**
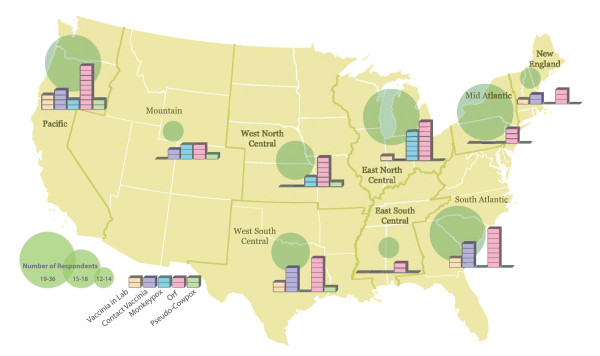
**Suspected poxvirus infections (omitting molluscum contagiosum) reported by region**. Each block in the bar charts represents one respondent. To permit more accurate comparisons of inter-regional variations, graduated green circles show the total number of respondents separated into three classes.

Respondents were also asked about the relative frequency with which they have ever encountered different poxvirus-associated illnesses in their practices. The majority (96.7%) had seen at least one case of molluscum contagiosum with 74.5% having seen five or more cases. 6% reported having seen at least 1 case of monkeypox, with six of these physicians practicing in the Midwest region (Figure 3). 4.7% reported having seen a case of vaccinia in a lab worker while 8.9% had seen a vaccinia infection in a social contact of a vaccinee. Respondents from each of the EIN regions reported having seen orf, with the largest number of those being in the Pacific, East North Central and South Atlantic region. Four respondents reported seeing a case of sealpox (one respondent in Providence, Halifax, and Sacramento and one in an unknown location). No one reported seeing a case of tanapox or a case of oral rabies vaccine (ORV) related human infection.

This survey provided an opportunity for public health practitioners to gain insight into physician approaches to evaluation, diagnosis and reporting of suspected poxvirus-associated infections. As a result we were able to identify potential knowledge gaps and deficits in the availability of useful resources to facilitate accurate case identification and management.

One of the weaknesses of this study was a low survey response rate. Several factors could account for this. Anecdotal suggestions are that many potential responders felt they were being "tested" rather than queried, and different professional groups had different response rates. Those who did not teach and those with less then 10 years of experience had a significantly lower response rate. Infectious disease physicians with less experience are probably less likely to have encountered some of these uncommon diseases and thereby are less likely to see the relevance of this query. Response rates also varied between regions, which could be due to differences in poxviruses seen in these regions.

While this sample may not be representative of the country's infectious disease physicians, it likely encompasses the best informed and the least apt to be dissuaded by lack of immediate knowledge. Respondents were able to suggest appropriate infection control measures, such as a combination of standard, contact and droplet precautions for monkeypox and standard precautions for orf, and pursued reasonable reporting mechanisms. A large proportion of respondents did, however, indicate that they would institute higher then necessary transmission precautions for the orf case scenario. We found that respondents were not necessarily aware of diagnostic tests available or where to find them. However, the majority of respondents did indicate they would order diagnostic testing for both case scenarios.

The public health community can play a greater role in reinforcing messages to health practitioners to address appropriate infection control procedures when dealing with suspected cases of poxvirus infection. They can also play an active role in disseminating information about new diagnostic tests (such as PCR and serologic tests). In response to findings from the survey, EIN members were provided a survey report summarizing CDC recommendations for the various scenarios [Additional file 2]. We also produced a fact sheet containing information pertinent to which diagnostic tests are currently available at CDC and elsewhere for etiologic determination of poxvirus-associated infection [Additional file 3]. The findings from this survey will also help guide us in the redesign of CDC's poxvirus website. We will make infection control practices, diagnostic capabilities, and reporting mechanisms more readily available on the website for physicians.

Poxviruses occur across the U.S. and around the world. Infectious disease physicians may not be the first clinicians to see patients with suspected poxvirus infections, but many will be asked to provide expert advice and consultation. Infectious disease physicians should be provided with the necessary tools to make well-informed decisions regarding suspected cases of poxvirus infections. This survey identified key areas in which public health practitioners can better serve physicians by focusing on education. These key areas include infection control practices and knowledge of various diagnostic tests available for poxviruses. Similar knowledge gaps exist for other relatively rare diseases that could become more common in an outbreak setting. This type of survey could also be helpful in bringing attention to those gaps and providing innovative ways to keep physicians better informed.

## Competing interests

The authors declare that they have no competing interests.

## Authors' contributions

CH, EL, MG and ID conceived of the survey, participated in its design and content, and assisted in the interpretation of results. CH did the statistical analysis, wrote the manuscript, and created the tables. CH and RL created the manuscript figures. SB and PP coordinated the survey design, distribution and collection of results. All authors reviewed, revised and approved the final manuscript.

## Supplementary Material

Additional file 1 **Poxvirus EIN survey**. A copy of the poxvirus survey sent to EIN members Click here for file

Additional file 2 **Final survey report**. EIN members were provided with this survey report summarizing CDC recommendations for the various scenarios Click here for file

Additional file 3 **Poxvirus diagnostic fact sheet**. Fact sheet containing information pertinent to which diagnostic tests are currently available at CDC and elsewhere for etiologic determination of poxvirus-associated infection Click here for file
