# Supplementary material for: Clinical experience, infection control practices and diagnostic algorithms for poxvirus infections - an Emerging Infections Network survey
Source: BMC Res Notes. 2010 Feb 25;3:46. doi: 10.1186/1756-0500-3-46 (PMC2841075; doi:10.1186/1756-0500-3-46)
Supplement: Additional file 1 — Poxvirus EIN survey. A copy of the poxvirus survey sent to EIN members [file 1756-0500-3-46-S1.DOC]

**EMERGING INFECTIONS NETWORK QUERY**

**Diagnostic Approaches to Suspected Poxvirus Infections**

Name:

**Case 1** (See picture 1):

A 23 year old male medical student arrives at your practice with several pustular skin lesions on upper and lower extremities (including the volar surfaces) and pronounced lymphadenopathy. He reported having missed classes several days previously due to feverishness as well as chills, backache and a general feeling of malaise. He has recently returned from the Democratic Republic of Congo where he, along with other medical students, volunteered at a provincial hospital. During his visit, he reports that he examined patients who presented to the clinic with an undiagnosed febrile rash illness.

**1. Which diagnostic procedures would you likely pursue?**

Clinical diagnosis alone *[Skip to question 2]*

**OR**

|  | Diagnostic lab utilized | | |
| --- | --- | --- | --- |
| *[Check all that apply]* | In-house/  local academia | State/CDC | Commercial reference lab |
| Swab for PCR/culture |  |  |  |
| Biopsy for histopathology/EM |  |  |  |
| Blood for serology |  |  |  |

**2. During evaluation of this patient, what precautionary measures would you take to prevent transmission?** *[Check all that apply]*

Routine precautions, as when evaluating any clinic patient

Contact precautions

Droplet transmission precautions

Airborne transmission precautions

**3. Given your suspicion as to the cause of this patient’s illness, which of the following agencies, if any, would be your first point of contact?** *[Select best answer]*

None; I do not suspect that this patient has a reportable disease

Local infection control

State/Local health department

CDC

Law enforcement (e.g., police or FBI)

Not sure; would depend on preliminary laboratory findings

Other, specify:

**Diagnostic Approaches to Suspected Poxvirus Infections** *(continued)*

**Case 2** (See picture 2):

A 42 year old male arrives at your practice with two large nonpruritic, painless vesicular lesions on his right thumb and forefinger. He reports no other symptoms. He works on a farm and he recently purchased several juvenile goats at an auction and noticed a few days later that they seemed to have ulcers on their oral mucosa. His hands have many abrasions due to his work on the farm.

**4. Which diagnostic procedures would you likely pursue?**

Clinical diagnosis alone *[Skip to question 5]*

**OR**

|  | Diagnostic lab utilized | | |
| --- | --- | --- | --- |
| *[Check all that apply]* | In-house/  local academia | State/CDC | Commercial reference lab |
| Swab for PCR/culture |  |  |  |
| Biopsy for histopathology/EM |  |  |  |
| Blood for serology |  |  |  |

**5. During evaluation of this patient, what precautionary measures would you take to prevent transmission?** *[Check all that apply]*

Routine precautions, as when evaluating any clinic patient

Contact precautions

Droplet transmission precautions

Airborne transmission precautions

**6. Given your suspicion as to the cause of this patient’s illness, which of the following agencies, if any, would be your first point of contact?** *[Select best answer]*

None; I do not suspect that this patient has a reportable disease

Local infection control

State/Local health department

CDC

Law enforcement (e.g., police or FBI)

Not sure; would depend on preliminary laboratory findings

Other, specify:

**Diagnostic Approaches to Suspected Poxvirus Infections** *(continued)*

**7. In your practice, have you ever seen cases that you suspected might be:**

|  | No | Not sure | Yes-**1** case | Yes, **2-4** cases | Yes, **≥5** cases |
| --- | --- | --- | --- | --- | --- |
| Contact vaccinia |  |  |  |  |  |
| …in lab workers |  |  |  |  |  |
| …in close contacts of vaccinees |  |  |  |  |  |
| …associated with oral rabies vaccine |  |  |  |  |  |
| Orf |  |  |  |  |  |
| Pseudocowpoxvirus infection (milker’s nodule) |  |  |  |  |  |
| Sealpox |  |  |  |  |  |
| Monkeypox |  |  |  |  |  |
| Tanapox |  |  |  |  |  |
| Molluscum contagiosum |  |  |  |  |  |
| Other poxvirus infections, specify: |  |  |  |  |  |

**8. When evaluating a patient with a disseminated vesiculopustular eruption, what patient criteria do you think are important in determining whether smallpox/monkeypox should be considered as a diagnosis, versus, for example chickenpox.**

*[Please list one criterion, i.e. “age”, in each box; you do not need to rank in order of importance]*

| 1. |  |
| --- | --- |
| 2. |  |
| 3. |  |
| 4. |  |

**9. What, if any, resources did you use to answer this query?**

Additional comments about poxviruses or this survey:

Is there anything you would like to report or do you have any questions for your network colleagues?

**Thank you for completing this survey!**
