# Supplementary material for: Clinical experience, infection control practices and diagnostic algorithms for poxvirus infections - an Emerging Infections Network survey
Source: BMC Res Notes. 2010 Feb 25;3:46. doi: 10.1186/1756-0500-3-46 (PMC2841075; doi:10.1186/1756-0500-3-46)
Supplement: Additional file 2 — Final survey report. EIN members were provided with this survey report summarizing CDC recommendations for the various scenarios [file 1756-0500-3-46-S2.DOC]

**IDSA Emerging Infections Network**

**Report for ‘Diagnostic Approaches to Suspected Poxvirus Infections’ Query**

**Overall response rate**: 211/1080 (**19.5%**) physicians responded from 2/20/07 to 3/22/07.

• Twenty-nine of these responses could not be linked with our member database, but these responses are included in the analyses below.

*Not all respondents answered all questions, so totals for individual questions vary.*

Geographical division (state) of practice:

New England (CT, ME, MA, RI, VT): 13 (6%)

Mid Atlantic (NJ, NY, PA): 27 (13%)

ENC (IN, IL, MI, OH, WI): 37 (18%)

WNC (IA, KS, MN, MO): 16 (7%)

S Atlantic (DC, FL, GA, MD, NC, SC, VA, WV): 35 (17%)

E S Central (AL, KY, MS, TN): 12 (6%)

W S Central (LA, OK, TX): 18 (9%)

Mountain (AZ, CO, NM, MT, UT, NV, WY): 14 (7%)

Pacific (CA, OR, WA): 35 (17%)

Canada (NS, ON) 2 (1%)

Practice type: Academic institution 105 (52%)

Private practice 83 (42%)

Other 12 (6%)

CASE 1: MONKEYPOX

**Question 1. Diagnostic procedures** [No. (%)]

• Clinical diagnosis alone (no labs) 8 (4%)

• Laboratory diagnostic procedures used 203 (96%)

*Table shows [No. (% of 203*)]

|  | In-house/local academia | State/CDC | Commercial reference lab |
| --- | --- | --- | --- |
| Swab for PCR/Culture | 61 (30) | 139 (68) | 25 (12) |
| Biopsy for histopath/EM | 99 (49) | 68 (33) | 8 (4) |
| Blood for serology | 37 (18) | 105 (52) | 36 (18) |

**Question 2**: **Precautionary measures** taken by members:

Routine precautions alone by 17 (8)

Contact precautions alone by 45 (21)

Droplet transmission precautions alone by 11 (5)

Airborne transmission precautions alone by 31 (15)

Droplet + Airborne by 1 (0.5)

Contact + Airborne by 32 (15)

Contact + Droplet by 14 (7)

Routine + Airborne by 1 (0.5)

Routine + Contact by 12 (6)

Contact + Droplet + Airborne by 11 (5)

Routine + Droplet + Airborne by 1 (0.5)

Routine + Contact + Airborne by 12 (6)

Routine + Contact + Droplet by 8 (4)

Routine + Contact + Droplet + Airborne by 12 (6)

*[CDC recommendations for possible monkeypox virus infection: ‘A combination of Standard, Contact, and Droplet Precautions should be applied in all health-care settings. In addition, because of the theoretical risk of airborne transmission, Airborne Precautions should be applied whenever possible.’]*

**Question 3**: Agency as **first point of contact**:

None by 5 (2)

Local infection control by 29 (14)

State/local health department by 126 (60)

CDC by 27 (13)

Law enforcement by 3 (1)

Not sure by 19 (9)

Other (not specified) by 1 (0.5)

*[Monkeypox is not a nationally notifiable infectious disease in the United States. State reporting requirements vary.]*

CASE 2: ORF

**Question 4. Diagnostic procedures**

• Clinical diagnosis alone (no labs) 55 (26%)

• Laboratory diagnostic procedures used 156 (74%)

Table shows [No. (% of 156)]

|  | In-house/local academia | State/CDC | Commercial reference lab |
| --- | --- | --- | --- |
| Swab for PCR/Culture | 70 (45) | 71 (46) | 26 (17) |
| Biopsy for histopath/EM | 67 (43) | 23 (15) | 6 (4) |
| Blood for serology | 17 (11) | 45 (29) | 28 (18) |

**Question 5**: **Precautionary measures** taken by members:

Routine precautions alone by 50 (24)

Contact precautions alone by 102 (49)

Droplet transmission precautions alone by 4 (2)

Airborne transmission precautions alone by 5 (2)

Contact + Airborne by 4 (2)

Contact + Droplet by 4 (2)

Routine + Contact by 32 (15)

Contact + Droplet + Airborne by 3 (1)

Routine + Contact + Droplet by 1 (0.5)

Routine + Contact + Droplet + Airborne by 2 (1)

*[CDC recommendations for possible orf virus infection: ‘Standard Precautions.’]*

**Question 6**: Agency as **first point of contact**:

None by 59 (29)

Local infection control by 30 (15)

State/local health department by 100 (49)

CDC by 5 (2)

Law enforcement by 3 (1)

Not sure by 10 (5)

*[Orf is not a national notifiable infectious disease in the United States. State reporting requirements vary.]*

**7. Seen suspected cases?** [No. (row %)]

|  | No | Not sure | Yes-**1** case | Yes, **2-4** cases | Yes, **≥5** cases |
| --- | --- | --- | --- | --- | --- |
| Contact vaccinia |  |  |  |  |  |
| …in lab workers | 196 (94) | 2 (1) | 8 (4) | 2 (1) | 0 |
| …in close contacts of vaccinees | 184 (89) | 3 (1) | 10 (5) | 7 (3) | 2 (1) |
| …associated with oral rabies vaccine | 206 (100) | 0 | 0 | 0 | 0 |
| Orf | 149 (73) | 3 (1) | 36 (18) | 14 (7) | 2 (1) |
| Pseudocowpoxvirus infection (milker’s nodule) | 196 (96) | 4 (2) | 3 (1) | 2 (1) | 0 |
| Sealpox | 204 (98) | 0 | 3 (1) | 1 (0.5) | 0 |
| Monkeypox | 190 (91) | 5 (2) | 7 (3) | 5 (2) | 1 (0.5) |
| Tanapox | 204 (100) | 0 | 0 | 0 | 0 |
| Molluscum contagiosum | 7 (3) | 0 | 8 (4) | 37 (18) | 158 (75) |
| Other poxvirus infections* | 152 (85) | 7 (4) | 4 (2) | 4 (2) | 12 (7) |

*Specified as: Chickenpox/varicella by 15, Vaccinia by 5, Rickettsialpox (nonviral) by 1, Smallpox by 2, Canarypox by 1, Herpes simplex 1 and 2 by 1.

**8. Patient criteria deemed important** in determining whether smallpox/monkeypox should be considered:

‘Travel’ or ‘travel history’ by 106 (50%)

‘Exposure history’ or ‘epidemiology’ by 75 (36)

Exposure history with multiple specific items listed by 9 (4)

‘Vaccination’ or ‘immunization history’ (sometimes including history of varicella) by 60 (28)

‘Distribution of lesions/rash’ by 55 (26)

‘Exposure history to animals’ or ‘contact with lab animals’ by 53 (25)

‘Stage of rash’ or ‘are lesions all at same stage’ by 50 (24)

‘Occupation’ (sometimes including specific jobs, e.g., lab researcher) by 43 (20)

‘Age’ by 31 (15)

‘Contact history’ or ‘exposure to sick contacts’ by 27 (13)

Characteristics of the lesions (e.g., superficial, size, morphology, pustular) by 27 (13)

‘Toxicity’ or ‘systemic illness’ by 26 (12)

‘Underlying immune status’ or ‘immunosuppressed’ by 15 (7)

‘VZV history’ or ‘varicella status’ or ‘varicella serology results’ by 13 (6)

Combination of lesion appearance and stage and size by 13 (6)

‘Prodrome’ or ‘incubation’ or ‘prodrome relative to rash’ by 9 (4)

Travel and exposure history (listed together) by 9 (4)

‘Known outbreaks’ or ‘other patients with known poxvirus infection’ by 9 (4)

‘Signs and symptoms’ by 8 (4)

‘Geography’ or ‘location’ by 7 (3)

‘History’ by 6 (3)

‘Lymphadenopathy’ by 4 (2)

‘Medical history’ by 4 (2)

‘Fever’ by 4 (2)

‘Physical exam features’ by 3 (1)

‘Epidemiologic risk factors’ by 3 (1)

‘Initial lab results’ or ‘biopsy’ by 2 (1)

‘Work, hobby, exposure hx (people, animal contacts’ by 2 (1)

Mentioned by 1 member each: ‘None come to mind’, ‘animal contact & ill human contacts’, ‘Common things happen commonly’, ‘Large vesicles on finger could be paronychia. So not culturing Strep or Staph’, ‘Intel on world terrorist activity’, ‘Drug exposure hx (drug rash)’, ‘Smallpox/monkeypox serology’ ‘Culture’.

**9. Additional resources** used to answer this query:

‘None’ (sometimes with an additional comment like ‘Now I know my level of ignorance’ or ‘my brain’ or ‘40 years experience’ or ‘probably should have’) by 146

Mandell, Douglas & Bennett’s Principles and Practice of Infectious Diseases by 11

CDC website by 7

AAP Red Book by 1

Hunter’s Tropical Medicine edited by Strickland by 2

UpToDate or eMedicine by 4

Google by 1

Krugman’s Infectious Diseases of Children by 1

Articles in NEJM, MMWR and Emerging Infectious Diseases, mentioned by 1 each

‘ID textbook’ by 2

‘Personal files’ by 1

Current Diagnosis and Treatment in Infectious Diseases by Wilson by 1
