# Supplementary material for: Clinical experience, infection control practices and diagnostic algorithms for poxvirus infections - an Emerging Infections Network survey
Source: BMC Res Notes. 2010 Feb 25;3:46. doi: 10.1186/1756-0500-3-46 (PMC2841075; doi:10.1186/1756-0500-3-46)
Supplement: Additional file 3 — Poxvirus diagnostic fact sheet. Fact sheet containing information pertinent to which diagnostic tests are currently available at CDC and elsewhere for etiologic determination of poxvirus-associated infection [file 1756-0500-3-46-S3.DOC]

CDC Poxvirus diagnostic specimen guide

| Specimen Type | Test | Orthopoxvirus  Variola, Vaccinia, Monkeypox, Cowpox | Parapoxvirus  Orf, Pseudocowpox, BPS | Yatapoxvirus  Tanapox |
| --- | --- | --- | --- | --- |
| Swab (in VTM or Dry)  Lesion, Lesion fluid, crust | PCR | x^1^ | x^2^ | x^3^ |
|  | Culture | x | x | x |
| Whole Blood (EDTA) | IgG | x^5^ | x^4^ |  |
|  | IgM | x^5^ | x^4^ |  |
|  | PCR | x^1,8^ |  | x^3,8^ |
| Serum  (or blood spun  in SS tube) | IgG | x^5^ | x^4^ |  |
|  | IgM | x^5^ | x^4^ |  |
| Tissue:  fresh or EM grid (shipped at 4^o^ C), or frozen | PCR | x^1^ | x^2^ | x^3^ |
|  | Culture | x | x | x |
|  | EM | x^6^ | x^7^ | x^6^ |
|  | IHC | x | x |  |
| Tissue  (Formalin fixed, paraffin block, fixed slides) | EM | x^6^ | x^7^ | x^6^ |
|  | IHC | x | x |  |
| Touch Prep Slides | PCR | x^1^ | x^2^ | x^3^ |
|  | Culture | x | x | x |

VTM: Viral Transport Media; PCR: Polymerase Chain Reaction; EM: Electron Microscopy; IHC: Immunohistochemistry.

1: Orthopox generic; Specific tests: Vaccinia, Monkeypox, Variola, Cowpox

2: Parapox generic, Orf specific, Bovine papular stomatitis specific, Pseudocowpox specific

3: Tanapox specific

4: Serologic testing for Orf is done by collaborating institutions

5: Serology is orthopox generic

6: Brick-shaped particles consistent with Orthopoxvirus, Molluscipoxvirus, or Yatapoxvirus

7: Ovoid virions consistent with Parapoxviruses

8: Appropriate for acute systemic infection

Note: A high quality digital photo helps assist in the diagnosis

Note: Consultation with your state communicable disease unit and state health laboratory is necessary before submission of specimens to CDC

For more information:

CSTE websites for state epidemiologists & infectious disease contacts: <http://www.cste.org/members/state_and_territorial_epi.asp>

<http://www.cste.org/Epipointofcontact/epidisplayID.asp>
